# Advances in neurochemical measurements: A review of biomarkers and devices for the development of closed-loop deep brain stimulation systems

**DOI:** 10.1515/revac-2020-0117

**Published:** 2020-12-31

**Authors:** Juan M. Rojas Cabrera, J. Blair Price, Aaron E. Rusheen, Abhinav Goyal, Danielle Jondal, Abhijeet S. Barath, Hojin Shin, Su-Youne Chang, Kevin E. Bennet, Charles D. Blaha, Kendall H. Lee, Yoonbae Oh

**Affiliations:** 1 Department of Neurosurgery Research, Mayo Clinic, Rochester, MN 55902, United States; 2 Medical Scientist Training Program, Mayo Clinic, Rochester, MN 55902, United States; 3 Division of Engineering, Mayo Clinic, Rochester, MN 55902, United States; 4 Department of Biomedical Engineering, Mayo Clinic, Rochester, MN, 55902, United States

**Keywords:** deep brain stimulation, neuromodulation, electrochemistry, electrophysiology, closed-loop, voltammetry

## Abstract

Neurochemical recording techniques have expanded our understanding of the pathophysiology of neurological disorders, as well as the mechanisms of action of treatment modalities like deep brain stimulation (DBS). DBS is used to treat diseases such as Parkinson’s disease, Tourette syndrome, and obsessive-compulsive disorder, among others. Although DBS is effective at alleviating symptoms related to these diseases and improving the quality of life of these patients, the mechanism of action of DBS is currently not fully understood. A leading hypothesis is that DBS modulates the electrical field potential by modifying neuronal firing frequencies to non-pathological rates thus providing therapeutic relief. To address this gap in knowledge, recent advances in electrochemical sensing techniques have given insight into the importance of neurotransmitters, such as dopamine, serotonin, glutamate, and adenosine, in disease pathophysiology. These studies have also highlighted their potential use in tandem with electrophysiology to serve as biomarkers in disease diagnosis and progression monitoring, as well as characterize response to treatment. Here, we provide an overview of disease-relevant neurotransmitters and their roles and implications as biomarkers, as well as innovations to the biosensors used to record these biomarkers. Furthermore, we discuss currently available neurochemical and electrophysiological recording devices, and discuss their viability to be implemented into the development of a closed-loop DBS system.

## 1 Introduction

Deep brain stimulation (DBS) is a neurosurgical procedure in which electrodes are implanted into specific anatomical targets within the brain, which are electrically stimulated to provide symptomatic relief in various movement disorders and neuropsychiatric conditions. DBS has yielded sustained benefits in remedying pathological symptoms and improving the quality of life for patients with neurological diseases including Parkinson’s disease (PD), essential tremor, Tourette syndrome (TS), and obsessive-compulsive disorder (OCD) [1–8]. In addition, with over 160,000 people worldwide having undergone the procedure, DBS has become one of the most common treatments for these and other neurological diseases [9]. Although DBS is an effective treatment modality, the underlying mechanisms mediating therapeutic responses are not yet well understood. A leading hypothesis describing the mechanism of action of DBS is that it restores aberrant neuronal firing to non-pathological rates via the application of high-frequency trains of electrical stimulation [10]. This stimulation also induces or inhibits the release of endogenous neurochemicals called neurotransmitters. These neurotransmitters, including dopamine, serotonin, adenosine, and glutamate, have been shown to play key roles in several neurological diseases [11–15]. For example, the degeneration of dopaminergic neurons in the substantia nigra, a structure within the mesencephalon (ventral midbrain), has been associated with abnormal motor symptoms, such as bradykinesia, resting tremor, and impaired posture and balance — hallmark symptoms of PD [16]. Thus, the use and measurement of these neurotransmitters as disease state biomarkers, when complemented with existing neurophysiological signal measurements, is not only a prerequisite to understanding the mechanisms of neuropsychiatric diseases, but also sets the foundation for the refinement of neuromodulation treatments.

To investigate the roles of neurotransmitters within pathophysiological states, researchers have relied on analyte sampling and physiological signal techniques such as microdialysis, voltammetry, and electrophysiology. Microdialysis involves inserting a small probe into tissue, wherein perfusate is run through the probe, allowing for the collection of soluble molecules from interstitial spaces. Further, given its broad reach, the scope of substances detectable by microdialysis is only limited by the analysis techniques available. However, this technique has several drawbacks that severely limit its viability for implementation in a close-loop system. Namely, it has a very slow time resolution (> 1 min, often around 10 min), and compared to other probes, the probe used in microdialysis causes significant damage to tissue integrity [17].

Thus, more feasible techniques for use in a closed-loop system, such as voltammetry and electrophysiology, may be suitable given their inherent advantages such as rapid time resolution and decreased tissue damage, among others. Voltammetry employs a range of neurochemical sensing techniques to monitor *in vitro* and *in vivo* neurochemical substances. Voltametric electrochemical techniques such as fast-scan cyclic voltammetry (FSCV) and multiple-cyclic square wave voltammetry (M-CSWV), in combination with carbon-fiber microelectrodes, have high sensitivity and selectivity during recordings while maintaining tissue integrity [18,19]. Nevertheless, FSCV and M-CSWV have features that significantly set one apart from the other. For example, FSCV is inherently limited to measuring the stimulation-evoked (phasic) release of neurotransmitters, while M-CSWV can measure resting tonic levels of neurotransmitters [18].

Electrophysiology techniques record membrane action potential, local field potentials, and oscillations of neurons to measure activities such as activation or inhibition. This is achieved using a small, high-impedance microelectrode that is inserted into the cell or area of interest, which then sends the membrane action potential signal via an amplifier to a base station where the signal is processed. Using electrophysiological data, researchers can assess neuronal firing characteristics, such as firing frequency or extract periodic data from local field potentials and characterize those signals. Examples of these techniques include electroencephalography (EEG) and electrocorticography (ECoG). EEG, widely used in neurological disease diagnosis and monitoring, measures synchronous neuronal firing over a large area of the brain and achieved via electrodes that are placed on the patient’s scalp [20]. Further, given its non-invasive nature and flexibility for electrode implantation, EEG provides researchers with wide range of applicability in their investigations. ECoG is a more invasive electrophysiological monitoring method that can utilize a wide array of sensors, ranging from subdural multichannel electrodes to depth electrodes capable of recording within deep brain structures [21]. ECoG provides increased spatiotemporal resolution compared to EEG and possesses minimal noise and artifact contamination that is commonly seen with muscle movements. Although an in-depth discussion on electrophysiological biomarkers is outside the scope of this review, it must be noted that conceptualization of a closed-loop DBS system will likely include a combination of voltammetry and electrophysiology sensing techniques. Thus, connections between the two methods will be made herein to discuss their viability and synergistic implementation in closed-loop DBS platforms.

Here, we provide an overview of common neurotransmitters and their key roles within neurological disease states. We also discuss currently available neurochemical and neurophysiological sensing hardware and software, the techniques utilized for recordings, and the expansion of recording capabilities by recent advances in biosensor modifications. Further, we assess the potential applications of novel neurochemical techniques in conjunction with available electrophysiological methods to measure biomarkers for use in closed-loop controlled DBS systems.

## 2 Biomarkers for closed-loop platform development

In recent years, options for the quantification of disease-relevant biomarkers, such as neurotransmitters or electrophysiological feedback, have expanded due to technological developments designed to enhance the sensitivity and selectivity of monitoring methods [22]. Necessarily, this expanded biomarker palette has tremendously improved researchers’ understanding of the dynamic, time-sensitive nature of neurological disease. Such understanding is crucial for the formation of analytics-informed treatment strategies, such as those employed by closed-loop controlled DBS systems.

**Figure 1:**
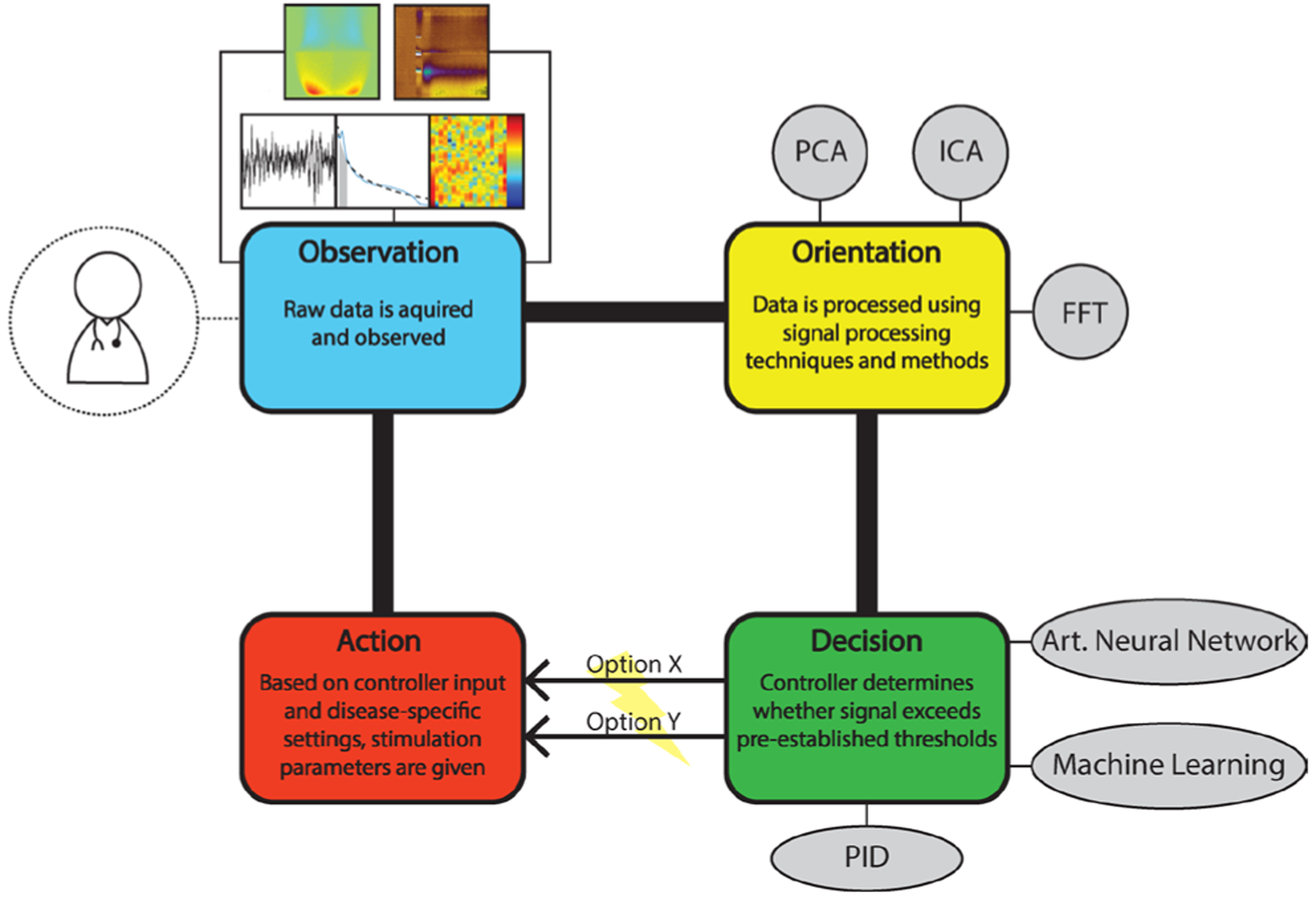
Depiction of a closed-loop DBS system utilizing the OODA loop framework. Abbreviations: PCA – principle component analysis; ICA – independent component analysis; FFT – fast Fourier transform; PID – proportional-integral-derivative.

An approach toward conceptualizing closed-loop DBS systems is to follow the structure of the Observation–Orientation–Decision–Action (OODA) loop. Originally developed by John Boyd for enhancing combat operations, the OODA loop is also highly applicable for understanding closed-loop controlled DBS systems (Figure 1). In this framework, the Observation phase encompasses the acquisition and measurement of electrochemical signals via voltammetry and amperometry, as well as electrophysiological signals, such as extracellular recording to ascertain single-unit activity or field potentials. These signals are then processed in the Orientation phase with an array of signal processing techniques and methods such as principle component analysis (PCA) and independent component analysis (ICA). Examples of signal processing within this phase are the conversion of voltametric data to neurotransmitter concentrations and electrophysiological time series data to frequency domains for oscillation period extraction. The next phase is the Decision phase which includes a controller system that receives the processed data and, based on pre-set thresholds specific to the disease and patient, selects the appropriate stimulation parameters to feed into the Action phase. Within the Action phase, the controller instructs the system to apply these stimulation parameters. An example of a controller is a proportional-integral-derivative (PID) controller, which is a basic mechanism that constantly calculates error from the information it processes, and proceeds to apply corrections to the system to reduce this error. However, more sophisticated controller systems such as artificial neural networks and machine learning based controlling systems are highly viable options for integration into this phase.

### 2.1 Dopamine

Dopamine (DA) is a neurotransmitter produced in the ventral tegmental area (VTA) and the substantia nigra with pathway trajectories to the nucleus accumbens and prefrontal cortex [25]. Like other catecholamine molecules (i.e. norepinephrine), dopamine is biosynthesized via the tyrosine hydroxylase enzyme and metabolized via monoamine oxidase and catechol-O-methyl transferase enzymes [25]. Dopamine plays an important role in the modulation of several neurological functions, including motor control, motivation, cognition, reward-seeking behavior, and prolactin release [26,27]. Accordingly, dopaminergic dysregulation is associated with several neurological and neuropsychiatric conditions such as Parkinson’s disease, addiction, mania, Tourette syndrome and schizophrenia [16,22,23,28,29]. For example, the leading hypothesis for Schizophrenia disease onset involves hyperactive dopaminergic activity in mesolimbic areas as well as the amygdala and prefrontal cortex, which are implicated in emotional processing and rational thinking [30]. Dopamine hyperactivity has also been postulated to influence the onset of motor tics, characteristic symptoms in patients with Tourette syndrome [13].

Clinical applications of dopamine monitoring have demonstrated promising results, as well as showcased the reliability of these techniques to monitor the neurotransmitter. For example, dopamine has been successfully recorded in both normal and disease states using microdialysis and voltammetry. Kilpatrick et al. (2010) successfully quantified a 30-minute baseline of dopamine *in situ* during a DBS surgery using microdialysis [31]. In 2011, Kishida et al demonstrated the first *in situ* voltametric measurement of real-time dopamine dynamics in the parkinsonian human brain using FSCV [32]. Furthermore, Kishida et al. (2016) demonstrated sub-second dopamine fluctuations during stock investment behavior tasks, finding that dopamine fluctuations were an integrated encoding of an individual’s evaluations of decision outcomes [33]. This provided clinical evidence for a neurochemical basis for feelings of regret or relief in response to decision.

Thus, developing measurement techniques to examine dopamine signaling in disorder-relevant neurocircuitry is a necessary step to further develop its use as a biomarker for closed-loop-controlled DBS applications. It is also apparent that although clinical monitoring of dopamine is technologically feasible, modifications to these sensing techniques must be made to achieve dopamine measurement over a long period (i.e. days, months, or years). Such modifications may include enhancing current or developing novel biosensors for chronic implantation whilst addressing common issues such as biofouling and material degradation, as well as refinement or development of a technique capable of long-term, reliable recordings. Once achieved, a sensitive closed-loop system could then be applied, for example, to Parkinson’s disease to monitor dopamine signaling in disease-relevant neural regions such as the subthalamic nucleus (STN). By additionally incorporating electrodes capable of neural signal sensing, this system could simultaneously monitor electrophysiological correlates of Parkinson’s disease, such as beta peaks, which are known to increase with bradykinesia [34]. The combined dynamic input from both neurochemical fluctuations and neural oscillatory activity may then be interpreted by the closed-loop system to modify stimulation parameters and judiciously apply stimulation to the STN according to the state- and time-sensitive needs of the patient (i.e. apply stimulation when dopamine levels or field potential frequencies fall outside of a pre-established threshold). Parameter modifications may be further refined through the application of machine learning and biomarker reports sent to the clinician, allowing for increased monitoring and ensuring treatment efficacy. In addition, dopamine signaling should be investigated chronically across multiple disease states to better delineate dopamine’s role in moderating disorder symptoms, further reinforcing the need for a long-term chronic recording system.

### 2.2 Serotonin

The serotonergic system is a common target for pharmacotherapy due to its roles in regulating emotion, mood, appetite, social behavior, and sleep [24,35]. Indeed, selective serotonin reuptake inhibitors (SSRIs) are the most commonly prescribed therapies to modulate serotonin signaling, thereby reducing symptoms in disorders such as depression or OCD [24,36]. Though the specific role serotonin plays in these disorders has not been fully characterized, the established effectiveness of drug-based modulation of serotonin neurotransmission to impart therapeutic effects warrants further investigation into its roles in mediating, moderating, or otherwise coordinating neurologic disease symptoms.

Serotonin has been shown to play a role in depression and depressive disorders like major depressive disorder (MDD). MDD is a mental illness that severely impacts social, behavioral, and physiological functions [37]. According to the National Institute of Mental Health, an estimated 7.1% of all adults in the United States experienced a major depressive episode during their lifetime [38]. Current research into the link between serotonin and depression has been promising. As mentioned earlier, the use of SSRI’s has been shown to have positive effects on patients with depression. However, studies seeking to causally link serotonin to depression by investigating mood changes after modulating neurotransmitter concentration have been inconclusive. These studies have shown that depleting serotonin’s precursor, tryptophan, can cause clinical depression characteristics to manifest themselves [39,40]. However, in what groups of people these manifestations occur vary. For example, people with no risk factors for depression had no significant response to a decrease in serotonin levels, whereas subjects with a history of depression had significant onsets of clinical symptoms of depression [37].

Serotonin was first recorded *in vivo* using FSCV in 1995 by Jackson et al. [41]. This group found that an N-shaped voltammogram waveform was necessary for serotonin detection using carbon-fiber electrodes. Following this milestone, multiple studies have successfully recorded serotonin in both animal and human brains. The latter was achieved by Moran et al. (2018) and involved recording serotonin using FSCV while the person performed an investment with reward and punishment outcomes depending on the magnitude and risk of their investment [12]. Researchers were able to see that when participants predicted the market negatively, serotonin levels fluctuated upwards, while positive predictions resulted in a downward fluctuation [12]. Interestingly, researchers were also able to predict increased cautious behavior in participants via increases in serotonin levels following events with negative outcomes, essentially showcasing that serotonin provided a protection mechanism from future losses. By employing serotonin concentrations as a biomarker in a closed-loop DBS system, we could further improve our understanding of the specific role that serotonin plays in disease onset and progress ion for depression, bipolar disorder, and other neuropsychiatric diseases, as well as more directly understand the clinical effects that modulation of serotonin has on mood.

### 2.3 Glutamate

Glutamate is an excitatory amino acid neurotransmitter involved in the promotion of neurogenesis and neuroplasticity. Glutamate achieves this in part through its activity at α-amino3-hydroxy-5-methyl-4-isoxazolepropionic acid (AMPA) and N-methyl-D-aspartate (NMDA) ionotropic glutamate receptors and subsequent promotion of brain-derived neurotrophic factor (BDNF) [42–44]. Esketamine, the S-enantiomer of racemic ketamine and non-competitive antagonist at the NMDA receptor, is an emerging pharmacological intervention for treatment-refractory forms of depression that facilitates neurogenesis in mood-related circuitry [45–47]. Though currently a challenging biomarker for long-term electrochemical recording, glutamate transfer functions have been developed [48] and the neurotransmitter may yet prove to be an option for guiding neuromodulatory therapeutics.

Real-time measurement of glutamate signaling has primarily been restricted to fixed potential amperometry and traditional cyclic voltammetry due to the non-electroactive nature of the molecule, precluding attempts at measurement via FSCV. Though measurable via fixed potential amperometry [49], glutamate measurement with this method requires isolation with high-performance liquid chromatography to remove interferents. However, coating platinum electrodes with glutamate oxidase – selectively converting glutamate to α-ketoglutarate and hydrogen peroxide – enables real-time faradaic measurement of glutamate indirectly via oxidative byproducts (hydrogen peroxide) as a proxy [48,50]. Investigations by Behrend et al. (2009) [48] for the use of glutamate as a biomarker in a closed-loop DBS system have found that extracellular glutamate levels in the STN of anesthetized rats were elevated by high frequency stimulation. A glutamate biosensor was placed in the STN of these rats, detecting glutamate fluctuations to dynamically control stimulation parameters. The resultant feedback-sensitive intermittent stimulation was able to maintain glutamate levels at any predefined level. However, the longevity of this method is limited due to fouling of the enzyme-coated platinum microelectrode, necessitating further technique development before long-term monitoring of glutamate levels can be achieved [50]. Nevertheless, advances in real-time *in vivo* glutamate measurement techniques may serve to significantly develop researchers’ understanding of the time-sensitive, phasic activities of glutamate signaling in neurological disease. This may, in time, also establish glutamate as a potential biomarker for stimulation parameter development and allow for further investigations into neuroplasticity, neurogenesis, and disease-relevant applications.

### 2.4 Adenosine

Adenosine, a purine nucleoside base and metabolite of adenosine triphosphate (ATP), is an inhibitor of excitatory synaptic transmission with a half-life of 8–15 s within the mammalian brain [51]. Studies have shown that following seizures, adenosine concentrations levels can increase up to 31-fold [51]. This immediate change following seizures served as a catalyst to investigate adenosine’s role in the event and was found to have neuroprotective attributes as well as a role in alleviating seizures. This hypothesis was further validated by studies where exogenous adenosine was administered and resulted in the termination of seizures in animal models of epilepsy [49,52–54]. Adenosine has also been successfully recorded in real time by Chang et al. (2012) [55] in essential tremor patients. This group utilized FSCV to record DBS-evoked adenosine changes within the brain and found that following implantation of the electrode, high-frequency stimulation of the ventral intermediate nucleus of the thalamus elicited adenosine release. These changes in adenosine were comparable to previous findings with fixed potential amperometry using enzyme-based electrodes. With these findings in mind, it is possible adenosine measurement may have applications for clinical use. FSCV-based neurochemical measurement technology to detect real-time changes in adenosine release, advanced by Venton and colleagues [56–58], substantially improves the utility and accessibility of clinical adenosine measurement. This provides an opportunity to measure adenosine fluctuations across disease states, such as during epileptiform events. Venton and colleagues also recently designed an automated algorithm to identify FSCV-measured transient changes in adenosine signaling [59]. This algorithm is useful for streamlining data analysis and may improve the utility of adenosine for clinical applications. With further development for long-term recording, adenosine could eventually be used as a biomarker to locate and even predict seizures within the brain. When coupled with a closed-loop DBS system, this could then provide time-sensitive neurostimulation, whether prophylactic or therapeutic, and give rise to a new treatment modality for patients with epilepsy and other seizure-related diseases.

### 2.5 Electrophysiological biomarkers

In addition to local field potentials, several other electrophysiological biomarkers have been proposed, including electroencephalographic potentials, electrocorticographic potentials, and action potentials [60]. However, it is worth noting that the employment of electrophysiological activity as biomarkers depends on the disease presentation. For example, electrocorticographic potentials have been established as an effective biomarker for seizure inhibition for epilepsy, while beta and gamma waves function as a proxy for bradykinesia [34]. As with neurochemical biomarkers, electrophysiological biomarkers should have high disease relevance and a high signal-to-noise ratio while being unaffected by external stimuli [61].

Ideally, effective disease monitoring will involve the integration of multiple biomarkers. This may include a combination of both neurochemical and electrophysiological measures. Recently, Vajari et al. (2018) developed a novel electrode employing a glassy carbon interface on a polyimide thin film wrapped around silicone rubber tubing [62]. This hybrid probe simultaneously detected neurotransmitter signaling and electrophysiological activity. Though requiring further validation and currently unsuited for chronic implantation, this is a step toward the development of measurement technologies and devices capable of examining multiple classes of biomarkers simultaneously. Such technology will prove beneficial for exploring brain structure and function as well as for integrating multimodal feedback for enhanced calibration of neurostimulation parameters. A more thorough discussion of the electrophysiological fundamentals can be found in recent reviews [60,63–65].

## 3 Biosensor innovations

### 3.1 Microelectrode modifications

Developments in the electrode manufacturing process have led to enhanced detection capabilities for adsorption-controlled substances. One such example is the use of a mixture of poly(3,4-ethylenedioxythiophene), commonly referred to as PEDOT, and Nafion, a perfluorinated ion-exchange polymer that is commonly electropolymerized onto the surface of carbon fibers. The use of PEDOT:Nafion coated electrodes *in vivo* and *in vitro* have led to significant improvements in neurochemical recordings, including increased sensitivity and selectivity for catecholamines such as dopamine. Vreeland et al. (2015) [66], for example, demonstrated that coating carbon-fiber microelectrodes using a 400 μM solution of EDOT, the pre-oxidation precursor of PEDOT, yielded 46 ± 13 nA/μM in response to *in vitro* dopamine deposition, compared to 13 ± 2 nA/μM for uncoated fibers. Others have shown that Nafion can similarly be used to improve electrode sensitivity to serotonin [67] and adenosine [68]. While increasing sensitivity to neurotransmitters, the use of the polymer coating has shown to mitigate biofouling during recordings, allowing for increased recording longevity, a quality essential for the development of chronic recording techniques.

Nevertheless, the benefits of PEDOT:Nafion coated electrodes are limited to electroactive neurotransmitters. For this reason, modifications to electrodes using biological components, like enzymes, are needed to record non-electroactive neurotransmitters. Since the introduction of the first enzyme-modified electrode for glucose measurement by Clark and Lyons in 1962 [69,70], novel microbiosensor modifications have expanded the capabilities of voltametric techniques. Chitosan, for example, is a polysaccharide extensively used due to its high affinity for protein and enzyme immobilization. When deposited onto carbon fibers, chitosan increases enzyme sensitivity and electrode stability with minimal protein disruption while capitalizing on FSCV’s electrochemical selectivity, thereby enhancing measurement capabilities for substances such as dopamine [71,72], serotonin [71], vitamin D_2_ [73], and glucose [74]. Such techniques have thus expanded the range of measurable substances by FSCV. For example, glutamate, a non-electroactive neurotransmitter, can be detected with fixed potential amperometry by coating platinum electrodes with glutamate oxidase [48]. This attracts local glutamate for oxidation, enabling subsequent quantification by the electrode.

The medium onto which neurotransmitters are adsorbed has also undergone extensive modification. Although carbon-fiber electrodes have become the standard for FSCV, other materials hold promise for voltammetric applications. For example, our group has investigated the use of boron-doped diamond electrodes [75]. Compared to carbon-fiber electrodes, boron-doped diamond electrodes are significantly more resilient, resisting surface degradation *in vivo*. Boron-doped diamond electrodes are also able to maintain neurochemical sensitivity to dopamine significantly longer than carbon-fiber electrodes, making them suitable for chronic recordings. However, though mechanically strong, the sensitivity of boron-doped diamond electrodes is inferior compared to conventional carbon-fiber electrodes. As such, further work is needed to develop an electrode capable of harnessing the strengths of both diamond and carbon fiber-based electrodes.

## 4 Devices for neurochemical and electrophysiological monitoring

**Figure 2:**
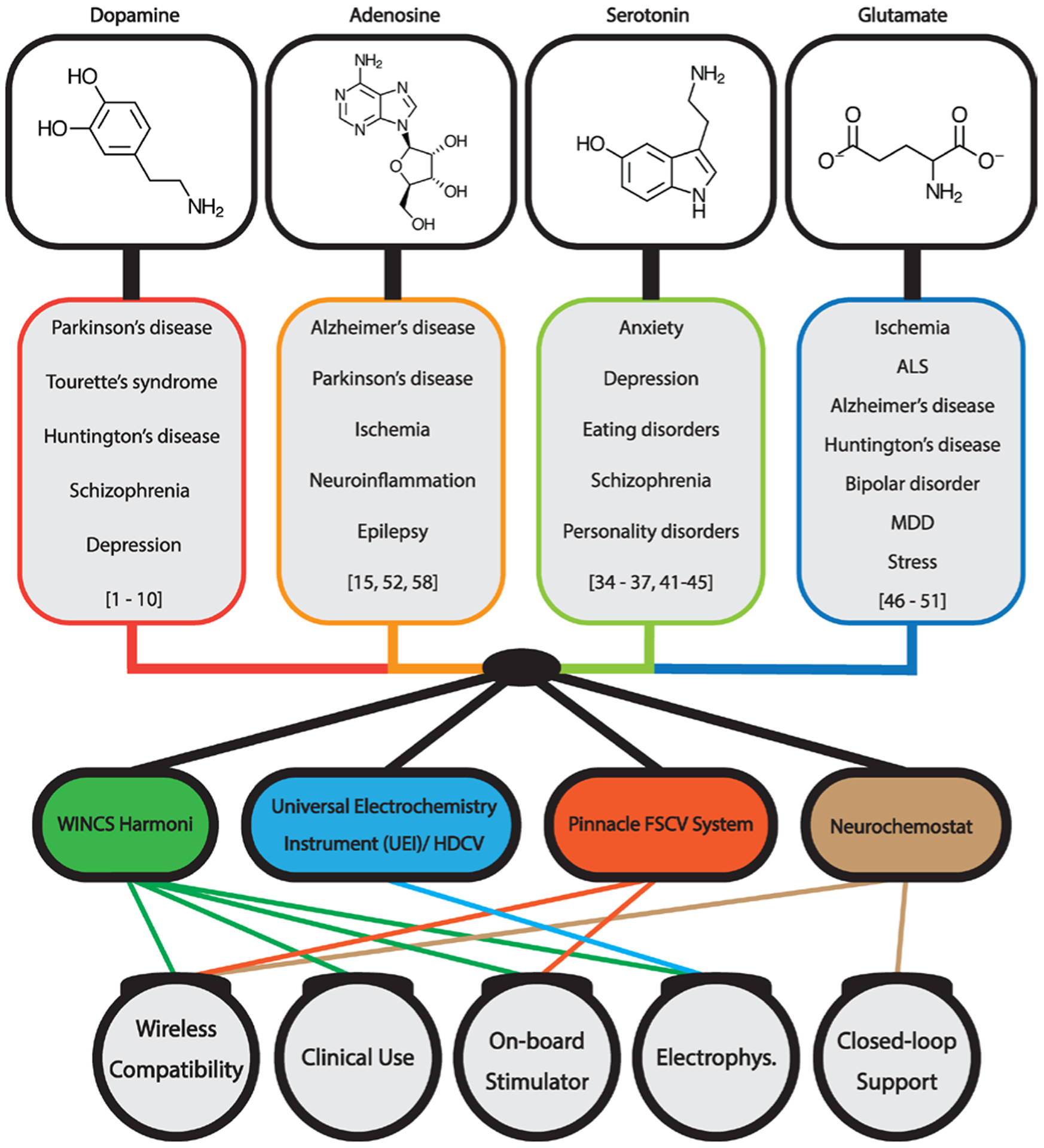
A schematic showcasing disease-relevant biomarkers and applicable neurochemical sensing devices. Abbreviations: MDD – major depressive disorder; HDCV – high-definition cyclic voltammetry; FSCV – fast-scan cyclic voltammetry.

A number of devices and software have been developed that enable the neurochemical measurement techniques described above, including electrophysiological monitoring. These systems variably provide accessible user interfaces and compactivity that facilitate their use and enable measurement technique flexibility through waveform modifications. Additionally, some of these systems showcase early steps in the development of technology capable of serving as a closed-loop DBS system via the combination of electrochemical recordings of neurochemicals and electrophysiological events, such as local field potentials, as biomarkers of neuropsychiatric disease states (Figure 2).

### 4.1 Universal Electrochemistry Instrument (UEI)

The Universal Electrochemistry Instrument (UEI) is a modular and configurable electrochemical and electrophysiological recording system developed at the University of North Carolina Chapel Hill in conjunction with Wightman and colleagues [76]. This system is arguably the most widespread and time-tested research instrument in present use. It is capable of four-channel electrochemical recording and two-channel electrophysiological recording. The system employs TarHeel CV, custom software permitting customizable waveform application and configurable electrical stimulation. The software is capable of synchronizing stimulation and recording for artifact mitigation.

Recently, however, Wightman and colleagues have introduced High-Definition Cyclic Voltammetry (HDCV) as an upgrade to the TarHeel CV software [77]. In addition to the features of TarHeel, HDCV expands its capabilities by improving its signal processing techniques, enables duel electrochemical and electrophysiological recording, and can support up to 16 simultaneous electrode recordings. Further, HDCV allows for increased waveform modifications by enabling simultaneous waveform application during FSCV recordings of any shape, further increasing the scope of reach for analyte and biomarker recordings. Thus, the UEI system, including both TarHeel CV and HDCV software, is capable of closed loop recording and stimulation. Nevertheless, although effective for research, the UEI system was not designed for human use.

### 4.2 Pinnacle FSCV System

Developed by Pinnacle Technology Inc., the Pinnacle neurochemical recording system utilizes FSCV to record neurotransmitters such as dopamine and norepinephrine [78]. This system comes in two varieties: a tethered and wireless system. The tethered version involves affixing a head stage to the animal’s skull, which is connected to a commutator directly above. The commutator is then connected to an FSCV interface box that directs and processes the data to the FSCV software. The wireless version of the Pinnacle system is much more compact and involves a head stage called the Rat Hat [78]. The Rat Hat contains the battery pack powering the system, as well as a wireless FSCV data acquisition and transmitting board which directs the data via Bluetooth to a paired USB dongle. Although custom waveforms can be uploaded and implemented easily, the FSCV system comes pre-programmed with three waveforms, which include triangle waveforms (−0.4 V to +1.1 V) for dopamine and norepinephrine detection, N-shaped waveforms (0.0 V to +1.1 V to −0.6 V) for serotonin, and sawhorse waveforms (−0.4 V to +1.3 V) for adenosine [78]. Multiple studies have demonstrated successful recordings *in vivo* with striatal recordings and electrical stimulation or pharmacological confirmation utilizing Pinnacle FSCV, making it a very well-established system [79,80]. However, although the Pinnacle system is compact and has wireless capabilities, like the UEI system, it is not designed for human use. Thus, further studies are needed to establish its feasibility for clinical experimentation. Additionally, given that the system solely utilizes FSCV, its recording capabilities are limited to phasic biomarker concentration detection.

### 4.3 Neurochemostat

Neurochemostat is the latest system on a chip (SoC) design from Mohseni and colleagues [81]. This system was purpose-built to permit closed-loop FSCV recording of dopamine and electrical microstimulation. The chip is an AMS 0.35 μm 2P/4M CMOS measuring 3.3 × 3.2 mm^2^. It contains a Neurochemostat design with an FSCV front-end and a stimulation back-end. FSCV is performed with a scan rate of 400 V/s at a 10 Hz recording rate, featuring digital signal processing with a decimation filter and concentration determination using principal component regression analysis. Stimulation is turned on or off based upon two user-defined gates. A successful demonstration was performed in rats with dorsal striatum recording and medial forebrain bundle stimulation [81]. Further development of this chip, including wireless control, control software, and a compact battery design may permit implantation studies in non-human primates and eventually humans.

### 4.4 Wireless Instantaneous Neurotransmitter Concentration Sensing (WINCS) System

The Mayo Clinic Neural Engineering Laboratories initially released the neurochemical sensing platform WINCS in 2009 [82]. This compact system was designed for use in the operating room and is capable of wireless, real-time neurotransmitter recording via Bluetooth using FSCV and fixed potential amperometry. In 2013, the Mayo Investigational Neurostimulator Control System (MINCS) was released, introducing an independent stimulating channel to the WINCS platform, thereby enabling simultaneous neurostimulation and neurochemical recording [83]. In 2017, the Mayo Clinic released the synchronized neuromodulatory and sensing platform WINCS Harmoni – a wireless, multichannel stimulating and sensing system for in-vivo neurochemical recordings across multiple anatomical targets [84,85]. WINCS Harmoni is also capable of applying either fixed potential amperometry or FSCV, therefore providing further flexibility in terms of experimental approach.

WINCS Harmoni is paired with the software platform WincsWare for wireless control and telemetry, enabling real-time data analysis and individualized stimulation parameter development [85]. WincsWare allows full control of the stimulation and sensing systems, featuring configurable parameter settings including pyramidal, N-Wave, and custom-designed FSCV scans with adjustable paired-pulse voltammetry [86] settings as well as fixed potential amperometry. This is particularly useful for clinicians interacting with distinct neurological diseases which, in order to sustain long-term therapeutic benefit, may require frequent adjustments of therapeutic parameters. As such, WINCS Harmoni may be applied to the development of closed-loop DBS systems. Together, this facilitates the development of highly sensitive therapeutic systems designed to interact with the patient’s disease state in real-time, enabling granular, fine-tuned control for the treatment of neurological and psychiatric diseases.

### 4.5 Neuropace® RNS

Originally introduced in 2013, NeuroPace announced the next generation RNS® System in 2018, which features improvements in battery life and memory capacity, compared to the initial NeuroPace RNS System [87,88]. The FDA-approved NeuroPace Next Gen RNS® system is designed to prevent epileptic seizures through early detection of aberrant electrocorticographic activity to direct real-time adjustable cortical stimulation of seizure onset zones. The readouts of cortical activity can be transmitted to clinicians, providing datasets for exploring long-term brain activity. A 2019 clinical study found that electrocorticographic activity measurement by this system, and its subsequent stimulation, was effective in reducing seizure incidence by 41.5% compared to 9.4% by sham-stimulated patients after five months of treatment [89]. Overall, the NeuroPace Next Gen RNS® system provides an example of an effective biomarker feedback-directed stimulation platform that intelligently provides therapeutic interventions only as the need arises, potentially reducing adverse treatment effects and prolonging the lifespan of the device.

Of note, this system showcases the longevity of the implanted electrode, a crucial advantage electrophysiological monitoring has over current electrochemical recordings. Electrodes like those used in the Neuropace RNS and other electrophysiological recording systems do not suffer from degradation in responsivity like that seen in biosensors used for electrochemical recording with techniques like FSCV and M-CSWV [90]. Chronic electrochemical monitoring must account for electrode longevity and associated changes to the recording of neurochemicals, as the sensitivity and selectivity of the implanted sensor may diminish over time. This, as presented earlier, necessitates the plethora of modifications required to mitigate these changes, as well as the complexities that come with striving for optimal, dependable signals. Thus, for now, electrophysiological biomarkers offer the most immediate means of developing a chronic closed-loop system.

### 4.6 Activa^®^ PC+S

In 2013, Medtronic announced the initial human implants of the Activa^®^ PC+S DBS system, which combined local field potential-based sensing with an adjustable stimulation algorithm [91]. Originally deployed for parkinsonian patients, the system was valuable in providing clinical data for a closed-loop DBS platform. Indeed, the Activa PC+S system is useful as an adaptive DBS platform; a 2018 study found that cortical narrowband gamma waves detected with the Activa PC+S system could direct stimulation parameters and patterns to reduce symptoms of dyskinesia [92]. This successfully demonstrated the feasibility of a sensing-and stimulating DBS platform, while reducing energy consumption by 38–45% compared to open-loop systems. Similarly, a 2015 study found that beta wave activity measured by the Activa PC+S system from the STN and globus pallidus was effective in directing stimulation parameters in a non-human primate model for Parkinson’s disease [93]. However, it should be noted that this system, as with other systems described herein, is not fully automated and therefore does not presently constitute a true closed-loop system.

## 5 Conclusions

The highly dynamic nature of neurological diseases presents a significant limitation to current open-loop DBS applications which do not incorporate biomarker-based feedback to monitor the disease state and adaptively modulate stimulation parameters. Variations within an individual’s condition, as well as between patients, highlight the need for a highly customizable and responsive interface to deliver individualized timely therapeutic neuromodulation. Therefore, the development of closed-loop DBS systems represents a major opportunity for innovation in the clinical application of stimulation-based therapies.

Advances in this field, however, are contingent on the development of novel electrochemical techniques and technologies. Such systems for enhanced neurochemical measurements and neuromodulation have made significant advances towards delineating the underlying mechanisms for various neurological disorders, as well as increased understanding of the mechanisms of DBS. Improvements in software, hardware and integrated sensing techniques have also expanded the scope and detail to which neurotransmitters can be recorded. Thus, the use and tracking of these neurotransmitters as biomarkers is logical given their role in neurological disorders. However, there remains a great deal of research and development to be done to bring these emerging devices and applications into widespread use in the operating room and for incorporation into the overall treatment plan for patients.
